# Fucoidans as Scientifically and Commercially Important Algal Polysaccharides

**DOI:** 10.3390/md19060284

**Published:** 2021-05-21

**Authors:** K. K. Asanka Sanjeewa, You-Jin Jeon

**Affiliations:** Department of Marine Life Science, Jeju National University, Jeju 690-756, Korea; asanka.sanjeewa001@gmail.com

## 1. Introduction

As a highly bioactive seaweed substance with many promising physiological activities, fucoidan has attracted attention from many industries all over the world. Even though fucoidans are a rich source of bioactive properties, the structural properties and bioactive mechanisms of fucoidans are poorly understood. Therefore, novel studies that either characterize the physical properties or biological activities of fucoidans will fill the knowledge gap between industrial applications and the scientific background of those applications.

Both purified and partially purified fucoidans isolated from brown seaweeds present high potential as preventative and therapeutic agents against a number of chronic diseases due to their anti-inflammatory, antioxidant, anticancer, neuroprotective, antiviral, antimicrobial, and anticoagulative properties.

This Special Issue is aimed at presenting updated information on well-documented studies of the structural characterization and major biological actions relevant for medical, cosmeceutical, and pharmaceutical applications that fucoidans isolated from brown seaweed can offer.

## 2. What Is Fucoidan

Fucoidan is a type of sulfated polysaccharide which contains a significant portion of L-fucose. Fucoidans are mainly extracted from brown seaweeds and one of the well-known bio-active polysaccharides collected from seaweeds [1]. Earlier Fucoidan was named as “fucoidin” when it was first separated from seaweeds by Kylin in 1913. According to the IUPAC rules correct term is fucoidan. However, some people still use fucosan, fucan, and sulfated fucan instead of fucoidan [2]. In recent years, fucoidan has been extensively studied due to its beneficial and interesting biological activities [3]. Specifically, fucoidan possess numerous bioactive properties such as, anti-inflammatory, immunomodulatory, antioxidant, anticoagulant, antivirus, anticancer, and gastric protective properties.

## 3. The Structure Characterization

With the effort of the scientific community, several fucoidans’ structures have been elucidated, and their biological activities have been identified [3]. The structure of fucoidans is complex in nature and it is very difficult to elucidate the general structure due to their heterogenous nature [4]. However, the majority of algal fucoidans consist of a linear backbone of random 1 → 3 or 1 → 4 linked α-L-fucopyranose residues periodically interrupted by other monosaccharides. Sulfate ester groups are arbitrarily substituted at 2, 3 and/or 4 positions of fucopyranose units, making it a highly heterogenous polymer [1,5].

## 4. How to Prepare

Fucoidan contents of brown seaweeds depend on a lot of factors such as variety, harvesting season, and maturity stage of seaweeds. In general, polysaccharides available in seaweeds can be extracted using water with or without some special enzymes, and separated by adding organic solvents [6]. However, extractions of cell wall polysaccharides are little bit difficult with the solvent extraction process. To extract cell wall polysaccharides such as fucoidan requires lyases in order to increase the extraction efficiency. Therefore, an enzyme-assisted extraction technique can be employed as an alternative method to enhance the extraction efficiency of fucoidan for industrial use [7].

In the article by Dörschmann, P. et al. [8], the authors enzymatically extracted *Saccharina latissima* fucoidan and isolated its low-molecular weight fraction. The results showed that the enzyme-treated fucoidans and the fractionated low-molecular-weight fucoidans are very promising for beneficial age-related macular degeneration-relevant biological activities. In order to modify fucoidans in molecular weights, fucoidans can be hydrolyzed by enzymes or phyco-chemical treatments and the resultant smaller fucoidans or fucoidan oligomers might improve bioactivities. In this Special Issue, a native fucoidan extracted from *Sargassum crassifolium* pretreated by single-screw extrusion was degraded by ascorbic acid or hydrogen peroxide. The lower-molecular-weight fucoidan prepared by ascorbic acid exhibits the highest cytotoxicity to A-549 cells and a strong ability to suppress Bcl-2 expression [9]. Tan, J. et al. [10] isolated fucoidan from *Saccharina japonica* and prepared its depolymerized fragment by oxidant degradation. The molecular weights of Fucoidan and its depolymerized fragment were 136 and 9.5 kDa, respectively. The low-molecular-weight fucoidan had higher renoprotective activity on adriamycin-induced nephrotic syndrome. On the other hand, Zayed, A. and Ulber, R. [11] mentioned in their review article that since a universal protocol for fucoidans production has not been established yet, all the currently used processes were presented and justified. The review article in the fucoidans field provided an updated overview regarding the different downstream processes, including pre-treatment, extraction, purification and enzymatic modification processes, and shows the recent non-traditional applications of fucoidans in relation to their characters.

## 5. Important Bioactivities

With the growing interest towards functional materials from natural sources, fucoidans isolated from different seaweeds have been scientifically and industrially studied aiming at assessing their potential biological activities such as anti-inflammatory, immunomodulatory, antioxidant, anticoagulant, antivirus, and anticancer properties [5,12]. Besides the cellular level activities, studies also focused on non-classical studies from fucoidans such as angiogenesis, treatment of intestinal diseases (inflammatory bowel disease and gastric ulcers), treatment of metabolic syndrome, and bone health supplements [13,14]. In this Special Issue, many articles provided bioactivities and physiological functionalities of fucoidans; for example, fucoidans of Moroccan brown seaweed act as an elicitor of natural defenses in date palm roots [15] and fucoidan from *Ascophyllum nodosum* suppresses postprandial hyperglycemia by inhibiting Na+/glucose cotransporter 1 activity [16]. It is well known that fucoidans show a protective effect against apoptosis [17] and ultraviolet B-induced photodamage [18]. On the other hand, one of the important bioactivities of fucoidans is an anticancer effect. Two articles in the Special Issue published anticancer activities of fucoidan via inducing apoptosis of cancer cells [19,20]. M. E. Reyes et al. reviewed brown seaweed fucoidan in cancer, in particular introduced implications in metastasis and drug resistance [21]. Typical bioactivities of fucoidans are antibacterial and antioxidant. Fucoidans in this Special Issue exhibited antibacterial effect against *Helicobacter pylori* infection [22] and antioxidant effect against oxidative stress [23]. Fucoidans can be effective on skin. Takahashi, M. et al. [24] have studied the improvement of psoriasis by alteration of the gut environment by oral administration of fucoidan. Furthermore, fucoidans were recently utilized to create a multilayer film with chitosan to bind fibroblast growth factor-2 for pharmaceutical-grade use [25].

## 6. Conclusions

Functional food is considered to be any food or food component that provides health benefits beyond basic nutrition. Recently, considerable attention has been directed by consumers towards functional ingredients from food, because of the lack of side effects. Compared with other polysaccharides, fucoidans are sulfated polysaccharides and are very active in their functions. In order to be commercially and widely available as functional food additives, a large number of fucoidans have been investigated in recent years.
